# In situ architecture of plasmodesmata in *Physcomitrium patens* resolved by cryo-electron tomography

**DOI:** 10.1038/s41477-026-02294-9

**Published:** 2026-05-14

**Authors:** Marcel Dickmanns, Matthias Pöge, Peng Xu, Sven Gombos, Zoe K. Barr, Manuel Miras, Jürgen M. Plitzko, Rüdiger Simon, Waltraud X. Schulze, Wolf B. Frommer, Wolfgang Baumeister

**Affiliations:** 1 Department of Molecular Structural Biology, Max Planck Institute of Biochemistry, Martinsried, Germany; 2 Faculty of Mathematics and Natural Sciences, Institute for Molecular Physiology, Heinrich Heine University Düsseldorf, Dusseldorf, Germany; 3 Department of Plant Systems Biology, University of Hohenheim, Stuttgart, Germany; 4 Faculty of Mathematics and Natural Sciences, Institute of Developmental Genetics, Heinrich Heine University Düsseldorf, Dusseldorf, Germany; 5 Research Group CryoEM Technology, Max Planck Institute of Biochemistry, Martinsried, Germany; 6 Cluster of Excellence on Plant Sciences, Heinrich Heine University, Dusseldorf, Germany; 7 Institute of Transformative Bio-Molecules, Nagoya University, Nagoya, Japan; 8 Present Address: Electron Microscopy Facility, Max Planck Institute of Molecular Cell Biology and Genetics, Dresden, Germany; 9 Present Address: Department of Stress Biology and Plant Pathology, CEBAS-CSIC, Murcia, Spain

**Keywords:** Plant cell biology, Cell biology, Plant physiology, Cryoelectron tomography

## Abstract

Plasmodesmata are nanoscopic channels that traverse plant cell walls, enabling direct intercellular exchange through membrane and cytosolic continuity. Although numerous plasmodesmal components have been identified, their molecular organization remains poorly defined. Here we used cryo-electron tomography to resolve the in situ architecture of plasmodesmata in *Physcomitrium patens* across tissues and physiological states. We show how callose-related cell wall remodelling shapes pore architecture to modulate permeability, including a previously undescribed fully sealed state, and resolve helical protein assemblies scaffolding the central, endoplasmic-reticulum-derived desmotubule. Candidate screening via proteomics and structure prediction indicates Multiple C2 Domain and Transmembrane Proteins (MCTPs) as key constituents of these assemblies. In this model, MCTPs tether the desmotubule to the plasma membrane, while their disordered linker regions with polyampholyte charge patterning may populate the cytosolic sleeve. These findings define core architectural features of plasmodesmata and provide a structural framework for understanding how membrane, protein and cell wall components coordinate intercellular connectivity in plants.

## Abstract (web summary)

Using cryo-electron tomography, the authors reveal how plasmodesmata (plant cytoplasmic bridges) are gated by cell wall remodelling and how helical protein assemblies scaffold internal membranes, with potential implications for molecular filtering.

## Main

Multicellular organisms rely on intercellular communication to coordinate growth, development and responses to environmental cues^1^. In plants, communication is complicated by the presence of a several-hundred-nanometre-thick cell wall. Direct molecular exchange across this barrier is facilitated by plasmodesmata (PD), nanoscopic intercellular channels with complex architecture and composition. PD traverse the cell wall and establish continuity between the cytosol, plasma membrane and endoplasmic reticulum (ER) of adjacent cells^2^. In animal tissues, direct cytoplasmic exchange largely occurs via transient protein channels that cluster at gap junctions. Cytoplasmic bridges containing shared membranes, however, are rare and typically restricted to the germline such as in the ring canals of insect germ cell cysts^1,3^. In contrast, PD are ubiquitous across both germline and somatic plant tissues, forming an almost organism-wide continuum of cells. This continuity necessitates the control of PD permeability to maintain cellular homeostasis and tissue identity^4,5^. Although PD are central to plant physiology and development, their molecular architecture and mechanisms governing permeability control remain incompletely understood. Proteomics-based approaches have identified hundreds of putative PD-associated proteins^6–13^, but an integrated molecular model is still lacking. Genetic dissection of individual components remains challenging, as loss-of-function mutants often exhibit severe systemic phenotypes or lethality^9,14,15^. In some cases, conditional expression systems or partial knockdowns have enabled functional studies, but genetic redundancy among PD-associated proteins further complicates analysis^16–18^. Fluorescence microscopy allows tracking of candidate protein localization and dynamics but lacks the spatial precision to resolve protein organization at PD and is prone to mislocalization artefacts from overexpression and fluorescent labelling. Hitherto, insight into PD ultrastructure has relied on electron microscopy^19,20^ and electron tomography^21,22^ of dehydrated, heavy-metal-stained and resin-embedded samples. These approaches have revealed some features of PD architecture and formation, yet the required treatments denature biomolecules and compromise molecular interpretation^23^. Recent advances in sample preparation and preservation have made cryo-electron tomography (cryo-ET) applicable to plant tissues^24^. In cryo-ET, three-dimensional imaging of pristinely preserved samples at macromolecular resolution allows the visualization of membrane interfaces and reveals the organization of protein complexes in their native cellular context. Here we used cryo-ET to resolve the architecture of PD in *Physcomitrium patens* and describe mechanisms controlling the permeability of plant cell–cell interfaces.

## Results

### In situ architecture of PD

**Fig. 1:**
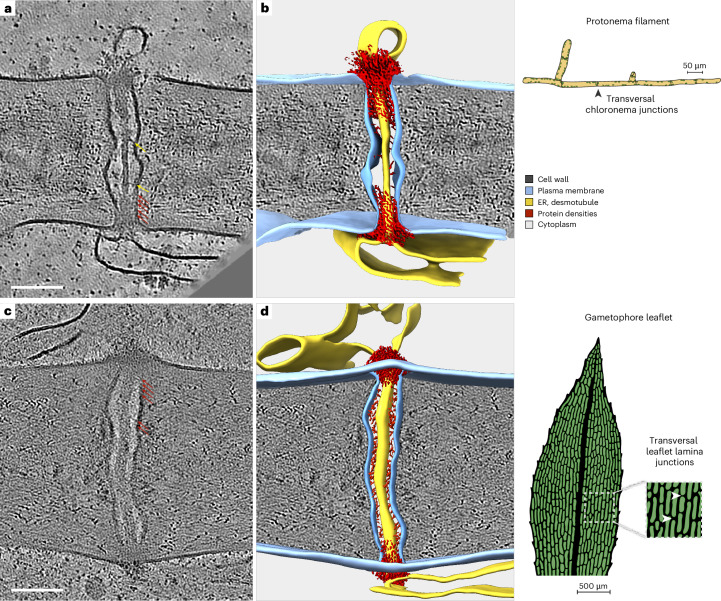
In situ architecture of PD in *P. patens* revealed by cryo-ET. **a**, 2D slice of a cryo-electron tomogram; cross-section of a cell wall between two cells within a protonema filament connected by a plasmodesma. Tethers connect wider desmotubule segments (red arrows) or narrower, central segments (yellow arrows) to the plasma membrane. **b**, 3D-rendered segmentation of tomographic volume in **a**. **c**, Slice through a plasmodesma connecting two gametophore leaflet lamina cells across their transversal cell wall interface. **d**, Segmentation of the tomographic volume in **c**. Scale bars, 100 nm.

**Extended Data Fig. 1:**
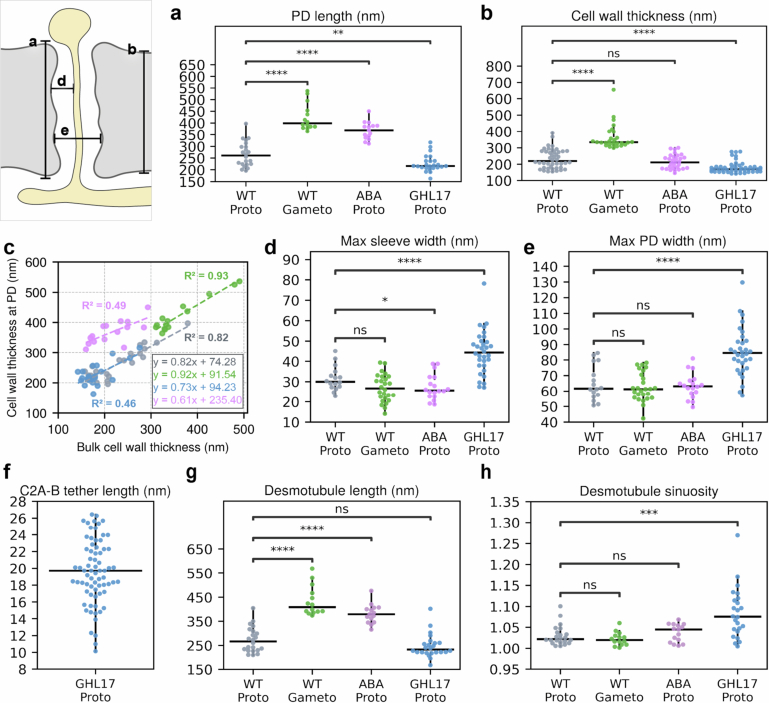
In situ measurements of plasmodesmata and cell wall dimensions. Quantification of plasmodesmata features in wild type protonemata (WT Proto, grey), wild type gametophores (WT Gameto, green), ABA-treated wild type protonemata (ABA Proto, magenta) and UBQ:GHL17_1 protonemata (GHL17 Proto, blue). Horizontal lines indicate medians; whiskers extend from the 25th and 75th percentiles to 1.5× the interquartile range. **a**, Plasmodesmata length. For sealed plasmodesmata in ABA-treated tissue, end-to-end length including callose plugs was measured. **b**, Bulk cell wall thickness measured in regions away from plasmodesmal channels, excluding bulged regions at plasmodesmata necks. Short-term ABA treatment (50 µM for 17 h) specifically induces bulging at plasmodesmata neck regions, whereas constitutive GHL17 overexpression broadly affects cell wall thickness at and away from plasmodesmata. **c**, Scatter plot of PD length versus bulk cell wall thickness with linear regression fits per data set (dashed lines); slopes and R^2^ (coefficient of determination) values indicate the degree and consistency of coupling. Lower R^2^ in ABA and GHL17 data reflects increased local cell wall heterogeneity compared to WT. **d**, Maximum width of cytoplasmic sleeve between desmotubule and plasma membrane. **e**, Maximum width of plasmodesmal channels, measured from plasma membrane to plasma membrane. **f**, Length of tethers originating from surfaces of desmotubule-coating assemblies and terminating at the plasma membrane. **g**, Total length of desmotubule contours. **h**, Sinuosity of desmotubules as an indicator of curvature. Desmotubules in GHL17 plasmodesmata have lengths similar to those in wild type but are accommodated within shorter channels, resulting in increased sinuosity. Two-sided Mann-Whitney-Wilcoxon tests (*P = 0.04; **P = 0.008; ***P = 0.0004; ****P ≤ 0.0001; ns, not significant); exact p-values are reported in Supplementary Data 1. Source data

While PD in land plants share a common evolutionary origin^25^, their architecture varies across species and tissues. In vascular plants, such as *Arabidopsis thaliana*, PD have diverse morphologies, including funnel-shaped and highly branched forms, reflecting tissue-specific adaptations^26–29^. PD in non-vascular plants such as the moss *P. patens* remain less studied^20,30^. To investigate their architecture, we recorded tomograms of cell–cell junctions in the two dominant tissues of *P. patens*, protonemata (*n* = 22 PD) and gametophores (*n* = 21 PD). Unlike the morphological diversity observed in vascular plants, we exclusively found unbranched PD in *P. patens*, exhibiting canonical features (Fig. 1). Each channel consists of a cell wall cavity lined by the plasma membrane and traversed by a central tubular extension of the ER, with a cytosol-filled intermembrane space, the cytosolic sleeve, between the ER and the plasma membrane (Fig. 1 and Supplementary Videos 1 and 2). In the wild type (WT), PD length correlates linearly with cell wall thickness, with thicker cell walls in gametophores necessitating longer PD (Extended Data Fig. 1). The neck regions directly adjacent to the apertures at each end of the plasmodesmal channel consistently represent the narrowest segments, assumed to impose structural constraints on molecular passage. The central, ER-derived desmotubule varies in diameter. In protonemata, desmotubules appeared wider at neck regions (24.1 ± 2.2 nm) and narrower at the centre of PD (10.5 ± 1.5 nm), while they remained uniformly wide (24.5 ± 3.1 nm) in gametophores. Desmotubules connect seamlessly to the cortical ERs of adjacent cells, regardless of cortical ER morphology, which can appear tubular (67%; top cells in Fig. 1; *n* = 61) with diameters substantially wider than the desmotubule inside PD or as sheets (33%; bottom cells in Fig. 1; *n* = 30) extending in parallel to the cell wall and cellular plasma membrane. Within the cytosolic sleeve, additional electron-dense material was observed. In protonemata, these likely proteinaceous features are most prominent at the neck regions, coinciding with wider desmotubule segments, but can also be detected along central desmotubule segments, where they are distributed sparsely. In gametophores, where desmotubules remain uniformly wide, these features are densely distributed along the entire length of PD. At narrow neck regions in both tissues, electron-dense material is most concentrated, obscuring desmotubules and the surrounding cytosolic sleeves.

### Impact of callose deposition and degradation on PD architecture

**Fig. 2:**
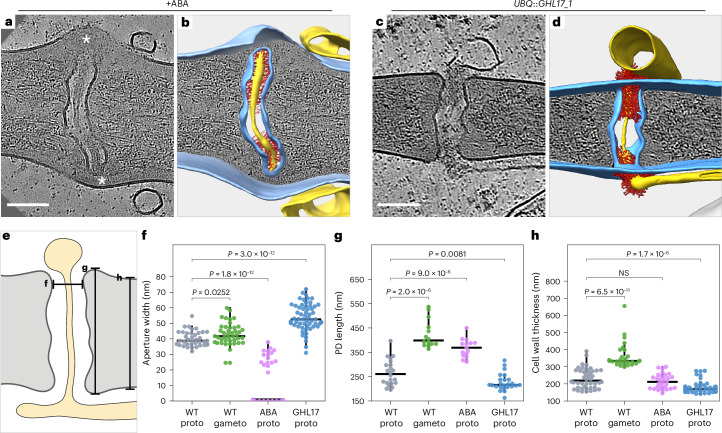
Deposition and degradation of the cell wall polymer callose gates PD. **a**, Incubating protonemata in 50 µM ABA for 17 h alters PD morphology. The asterisks mark putative callose deposits, disconnecting PD from cellular membranes and cytosol. **b**, Segmentation of tomographic volume in **a**. **c**, Overexpression of the callose-degrading enzyme GHL17 results in shorter and wider PD. **d**, Segmentation of tomographic volume in **c**. **e**–**h**, Effects of callose levels on PD architecture. The horizontal lines indicate medians; the whiskers extend from the 25th and 75th percentiles to 1.5× the interquartile range. Two-sided Mann–Whitney–Wilcoxon tests were used to determine significance. Exact *P* values are indicated in the panels. The measurements are from *n* = 22 (WT proto), 21 (WT gameto), 20 (ABA proto) and 38 (GHL17 proto) PD from 3 independently grown and prepared cultures per condition. The panels show the measurement positions (**e**); the aperture diameter at neck regions, representing the narrowest positions within PD (**f**); PD length (**g**); and bulk cell wall thickness (**h**). NS, not significant. Scale bars, 100 nm. Source data

**Extended Data Fig. 2:**
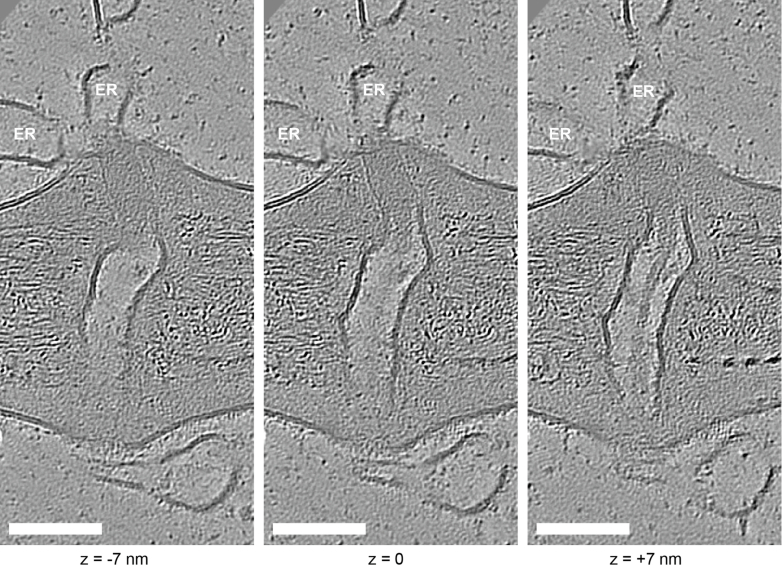
Tomographic z-slices of a plasmodesmal aperture after ABA-treatment. Localized cell wall bulging is observed at the neck regions without detectable membrane severing. Electron-dense material is accumulated within the cytosolic sleeve, obscuring parts of the desmotubule.

**Extended Data Fig. 3:**
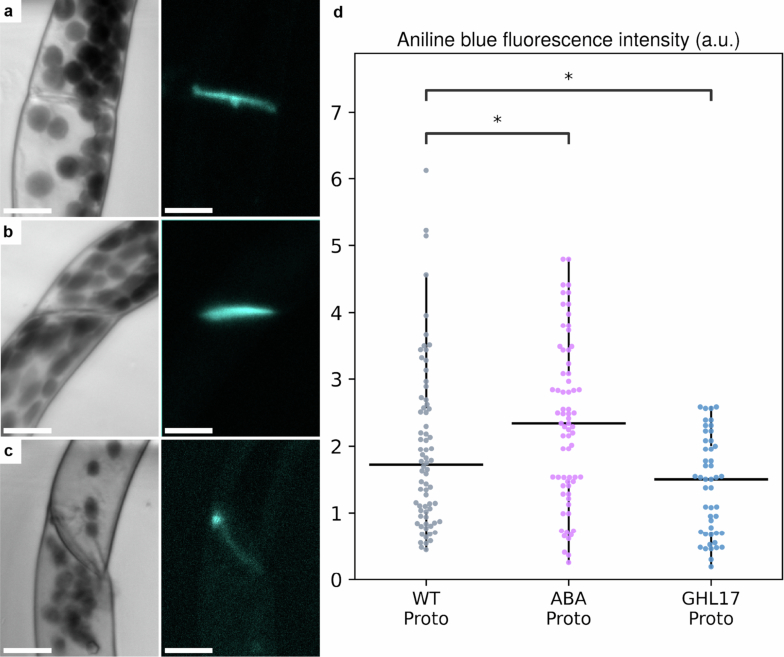
Callose quantification by aniline blue staining in *P. patens*. **a-c**, Z-projections of protonemata cell-cell interfaces in transmitted light (left) and fluorescence of aniline blue (right, cyan) marking callose. **a**, Wild type protonemata. **b**, ABA-treated wild type protonemata. **c**, UBQ:GHL17_1 protonemata. **d**, Quantification of aniline blue fluorescence intensity. Horizontal lines indicate medians; whiskers extend from the 25th and 75th percentiles to 1.5× the interquartile range; two-sided Mann-Whitney-Wilcoxon test; P = 0.0267 (WT Proto vs. ABA Proto), P = 0.0219 (WT Proto vs. GHL17 Proto); measurements from n = 73 (WT Proto), n = 69 (ABA Proto), and n = 48 (GHL17 Proto) cell-cell interfaces from 3 independent replicates respectively. Scale bars: 10 µm. Source data

To balance intercellular exchange and cellular homeostasis, plants dynamically regulate PD permeability. A key mechanism involves callose, a β-1,3-glucan cell wall polymer, which is deposited around neck regions by callose synthases and degraded by β-1,3-glucanases^17,31–33^. Synthesis and degradation either narrow or widen PD apertures, modulating permeability in response to developmental and environmental cues^34^. Under conditions such as water scarcity, pathogen infection or winter dormancy, plants induce callose synthesis via the stress-response hormone abscisic acid (ABA)^35–38^. Unlike cellulose, which forms a dense fibrillar meshwork in the bulk cell wall, callose-rich deposits are structurally more amorphous^39^. Cryo-ET of ABA-treated protonemata revealed large, granulated deposits of cell wall material around PD neck regions (Fig. 2a,b). While neck regions generally became constricted after ABA treatment (Extended Data Fig. 2), 45% of PD apertures (15 of *n* = 33 from 20 PD) exhibited a more severe phenotype, where deposition led to complete abscission of cellular plasma membranes, ER and cytosolic sleeves (Supplementary Video 3). The cortical ER remained tethered to the plasma membrane near the former connection site to the desmotubule. These drastic changes in PD architecture provide a structural explanation for the ABA-induced reduction in intercellular trafficking reported in *P. patens*^40^. In contrast, we previously found that overexpression of the callose-degrading β-1,3-glucanase *Pp*GHL17_1 increases the permeability of cell–cell interfaces^11^. To determine whether and how increased permeability correlates with changes in PD architecture, we performed cryo-ET on a glucanase-overexpressing line, hereafter referred to as GHL17 (Fig. 2c,d and Supplementary Video 4; *n* = 38 PD). We found PD in the protonemata of GHL17 to be shorter and wider, not only around neck regions but along the entire channel (Fig. 2e–h and Extended Data Fig. 1). In accordance with PD conductance models predicting lower resistance and thus increased molecular flow in wider and shorter PD^28,41^, these architectural changes explain the enhanced cell–cell permeability phenotype in GHL17. Callose quantification by aniline blue staining indicated lower callose levels at GHL17 cell–cell interfaces than in the WT and elevated callose levels in ABA-treated WT tissue (Extended Data Fig. 3). Cell wall material with a granulated texture seen at PD necks in tomograms was weakly pronounced in the WT and GHL17 and expanded upon ABA treatment, supporting the interpretation that granulated textures correspond to callose-rich deposits.

### Helical protein assemblies coat desmotubules at PD neck regions

**Fig. 3:**
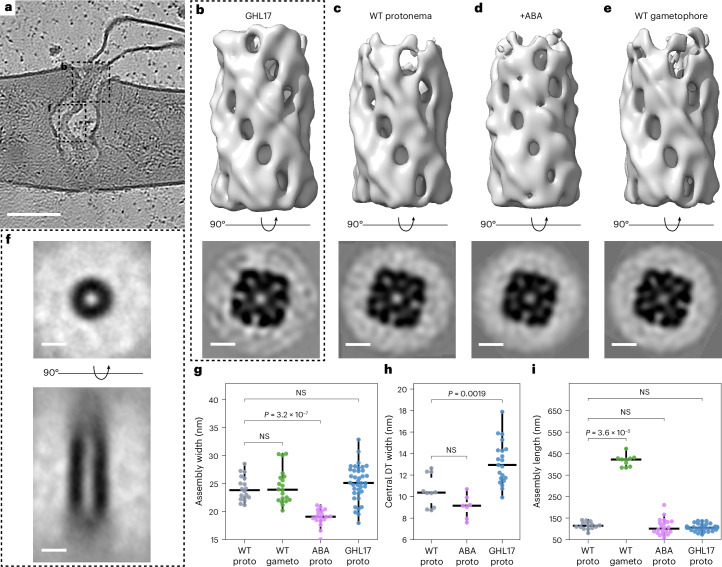
Helical desmotubule-coating assemblies are core architectural features of PD. **a**, Tomographic slice through a plasmodesma in a GHL17 protonema. The dashed rectangles indicate regions where subtomograms for averaging along the desmotubule were extracted. **b**–**e**, Resolved desmotubule-coating assemblies in GHL17 protonemata (**b**), WT protonemata (**c**), ABA-treated WT protonemata (**d**) and WT gametophores (**e**). Top, side views of 3D-rendered assemblies. Bottom, 2D cross-sections through assemblies. **f**, Transverse (top) and longitudinal (bottom) cross-sections of the averaged central desmotubule segment in protonemata, where assemblies are absent. **g**–**i**, In situ measurements of assemblies. The horizontal lines indicate medians; the whiskers extend from the 25th and 75th percentiles to 1.5× the interquartile range. Two-sided Mann–Whitney–Wilcoxon tests were used to determine significance. Exact *P* values are indicated in the panels. The measurements are from *n* = 22 (WT proto), 21 (WT gameto), 20 (ABA proto) and 38 (GHL17 proto) PD from 3 independently grown and prepared cultures per condition. The panels show the assembly width (**g**), the diameter of the central desmotubule (DT) segment in protonemata (**h**) and the assembly length (**i**). Scale bars, 100 nm (**a**); 10 nm (**b**–**f**). Source data

**Extended Data Fig. 4:**
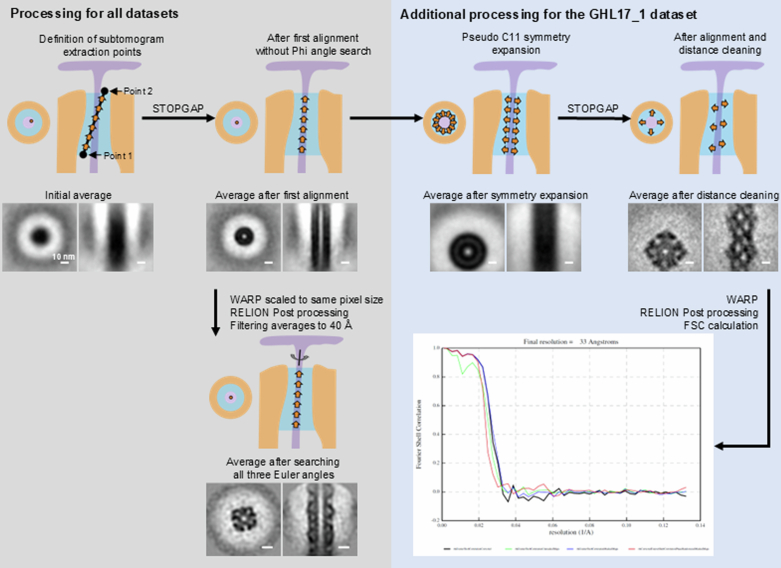
Subtomogram averaging workflow. For each alignment step, the top panel shows a schematic representation, and the bottom panel displays an xy cross-section and an xz-plane side view through the center of the resulting subtomogram average. Subtomogram extraction points are indicated by orange arrowheads. The direction of each arrow represents the subtomogram z-axis as defined by the Euler angles θ (Theta) and ψ (Psi). Scale bars: 10 nm.

Benefiting from wider PD in GHL17 protonemata, we identified periodic densities decorating all desmotubules at neck regions, while central segments lacked such features (Fig. 3a). These coatings cover approximately the first third of the desmotubule on both sides of PD in protonemata. In GHL17, the electron-dense material that had partially obscured desmotubules at neck regions of WT PD now appeared to extend from the desmotubule coatings towards the plasma membrane. Prompted by the periodic appearance of the coatings, we performed subtomogram averaging along the desmotubule (Extended Data Fig. 4). At neck regions, we resolved assemblies of structurally repetitive units winding around the desmotubule and anchored to the membrane, forming a helical lattice composed of four intertwined wraps (Fig. 3b). The electron-dense material extending from the surface of the assemblies towards the plasma membrane escaped structure determination by averaging, consistent with a flexible arrangement. Similar helical assemblies were recovered by averaging desmotubules in WT protonemata (Fig. 3c), ABA-treated protonemata (Fig. 3d) and gametophores (Fig. 3e), indicating that they are defining architectural features of PD in *P. patens*, consistently present across different cell types and physiological conditions. Averaging of central desmotubule segments confirmed that these ordered coatings are restricted to PD neck regions in protonemata (Fig. 3f). Notably, we measured slightly reduced diameters of desmotubule coats under ABA treatment (Fig. 3g), whereas GHL17 overexpression was associated with a significantly wider central, coat-free desmotubule region (Fig. 3h). Besides differences in desmotubule diameter, assembly lengths varied across tissues and conditions as well as between individual PD (Fig. 3i). In gametophores, the entire desmotubule is coated, whereas in protonemata, assemblies are confined to the neck regions. The variability in length and overall periodic structure suggests a modular organization assembled from recurring protein units.

### MCTPs are the likely constituents of the desmotubule coat

**Fig. 4:**
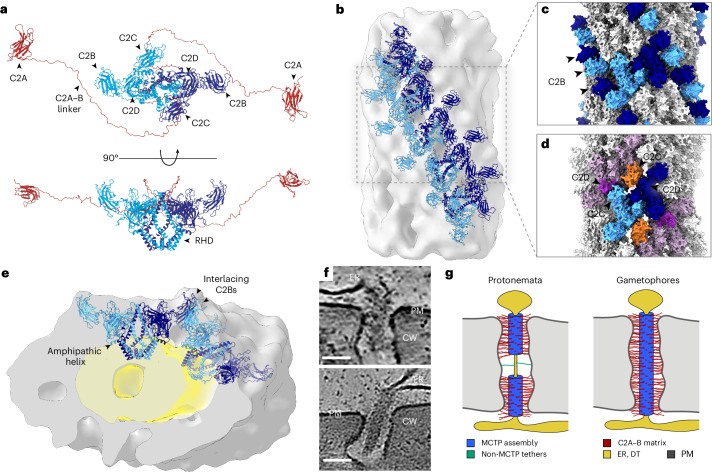
Arrangement of *Pp*MCTPs in the desmotubule-coating assembly. **a**, Structure prediction of full-length, dimeric *Pp*MCTP2. N-terminal C2A domains connect through flexible linkers (red) to the dimerizing core (monomers in light and dark blue). Four central α-helices form the dimer interface. RHDs anchor the dimer to the ER-derived desmotubule membrane. The C2B, C2C and C2D domains are accessible for protein–protein interactions. **b**, Subtomogram average of the desmotubule-coating assembly with docked models of dimeric *Pp*MCTP (blue), tiled along one of four helical wraps. Flexible linkers and C2As are not resolved in the average. **c**, Surface rendering of the assembly. C2B domains of dimers from adjacent helical wraps interlace (light and dark blue). **d**, Dimers within the same helical wrap contact each other via C2C and C2D domains. **e**, Angled cross-section of the assembly (grey) with two docked dimers (blue) and the desmotubule membrane region (yellow). **f**, Magnified views of *Pp*MCTP assemblies in situ. CW, cell wall; PM, plasma membrane. Scale bars, 50 nm. **g**, Schematic of desmotubule organization in protonemata and gametophore tissues. MCTP assemblies (blue) coat desmotubules, with C2A domains and C2A–B linkers (red) extending towards the plasma membrane. In protonemata, the central desmotubule regions are not coated by MCTP assemblies and have occasional tethers extending to the plasma membrane (green). In gametophores, desmotubules are fully coated by MCTP assemblies.

**Extended Data Fig. 5:**
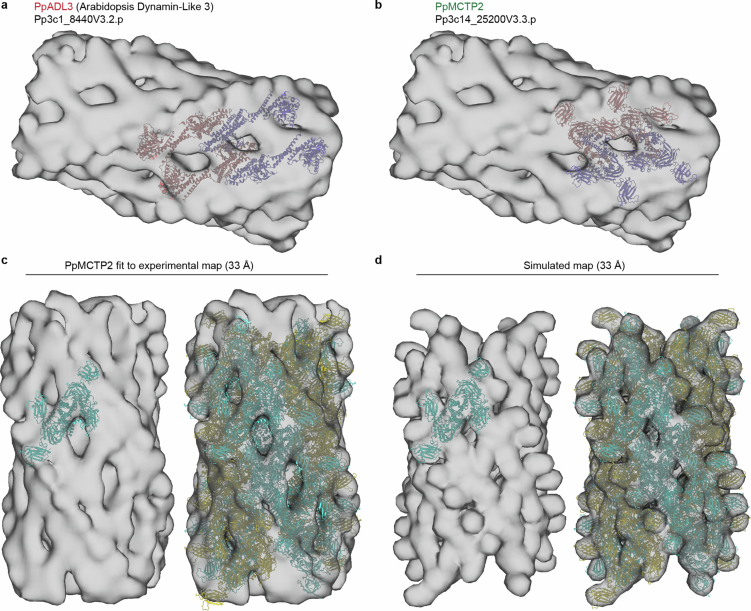
Fitting of predicted candidate protein structures into the experimental density map. To evaluate potential components of the desmotubule-associated coat, we tested candidates identified in PD-enriched proteomes. Dynamin-related proteins emerged as plausible candidates based on their property to assemble into large complexes associated with ER and their membrane tubulating function^82^. Such assemblies are formed by repeating dynamin dimers. **a**, Two predicted *Pp*ADL3 dimers were fitted into the experimental density map of the desmotubule-coating assembly (monomers in pink and purple). Although the dimer models span the assembly lumen, the fit leaves substantial regions unaccounted for and fails to recapitulate structural features. **b**, In contrast, *Pp*MCTP2 dimers align closely with the experimental map, matching its local geometry. **c**, A full molecular model was generated by tiling *Pp*MCTP2 dimeric core units along the resolved coat. Dimers colored in cyan and yellow, alternating colors between helical wraps. **d**, From the full molecular model based on the experimental map, a corresponding synthetic map was generated. Filtered to 33 Å resolution, the synthetic map closely resembled the experimental density map. The cross-correlation coefficient between synthetic and experimental map is 0.92, indicating a good overall agreement between model and experiment.

**Extended Data Fig. 6:**
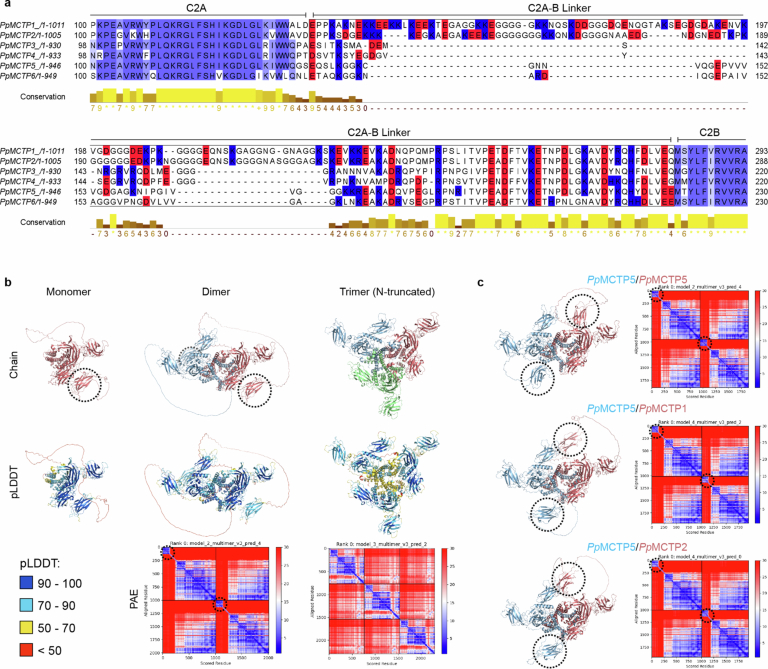
Sequence and structural analysis of *Pp*MCTPs. **a**, Alignment of C2A-B linker domains. While C2 domains are highly conserved, the linker domains vary in length and residue composition. These linkers are glycine-rich polyampholytes, with evenly distributed charges (blue: positive, red: negative). This composition suggests high solubility and flexibility, with a propensity for unspecific intra or intermolecular interactions. Identical residues in C2A and C2B are colored in blue. **b**, AlphaFold2-predicted structures of monomeric, dimeric, and trimeric *Pp*MCTP2 with corresponding model confidence metrics. Dotted circles mark positionally flexible C2A domains. While the predicted local distance difference test scores (pLDDT) within C2A domains are high, indicating well-defined local structure, the predicted aligned error (PAE) between the C2A domain and the rest of the protein exceeds 30 Å, suggesting that the domain is positionally flexible while maintaining overall domain integrity. In contrast, C2A-B linker domains exhibit low pLDDT (<50) and high PAE (>30 Å), supporting intrinsic flexibility. Central helices and RHDs show increased pLDDT in the dimer compared to the monomer. In trimeric models, both pLDDT and PAE indicate reduced model confidence at oligomerization interfaces, supporting the dimer model as the favorable oligomeric arrangement. For trimer predictions, the flexible N-terminus was truncated to reduce compute, as it does not participate in oligomer contacts. **c**, Structure predictions reveal no substantial differences between homo- and hetero-*Pp*MCTP dimers. Besides the positional variability of C2A domains (dotted circles) and C2A-B linker domains, the predicted conformations are nearly identical and no apparent shift in confidence metrics is observed. Combined, sequence analysis and structure prediction suggest that both homo- and heterodimeric pairings of all six *Pp*MCTPs are biochemically and structurally plausible.

**Extended Data Fig. 7:**
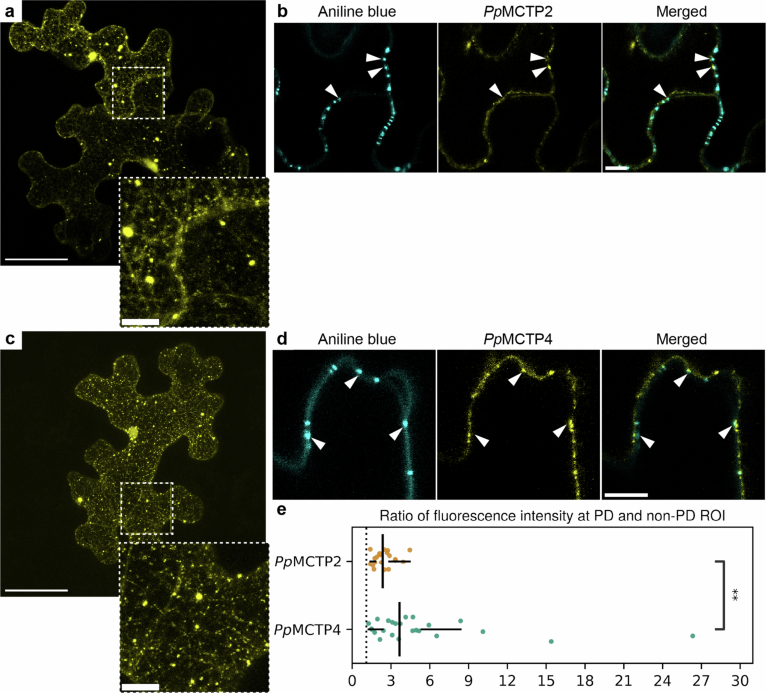
In vivo localization of *Pp*MCTPs in *N. benthamiana*. **a,c**, Maximum intensity z-projections showing (**a**) *Pp*MCTP2 and (**c**) *Pp*MCTP4 localization in epidermis cells. Dashed rectangles indicate region magnified in inset. **b,d**, Dotted localization patterns of the plasmodesmal marker aniline blue (cyan) and mVenus-*Pp*MCTPs (yellow) in single z-slices focused at the cell periphery. White triangles exemplify co-localization. **e**, PD index quantification of *Pp*MCTPs. Proteins with a PD index > 1.1 (dotted line) are considered enriched at plasmodesmata^11^. Vertical continuous lines indicate medians; whiskers extend from the 25th and 75th percentiles to 1.5× the interquartile range; two-sided Mann-Whitney-Wilcoxon test (**P = 0.0055); n = 23 (*Pp*MCTP2) and n = 24 (*Pp*MCTP4) from 3 independent replicates respectively. Scale bars: (**a,c**) 500 µm, inset 10 µm; (**b,d**) 10 µm. Source data

**Extended Data Fig. 8:**
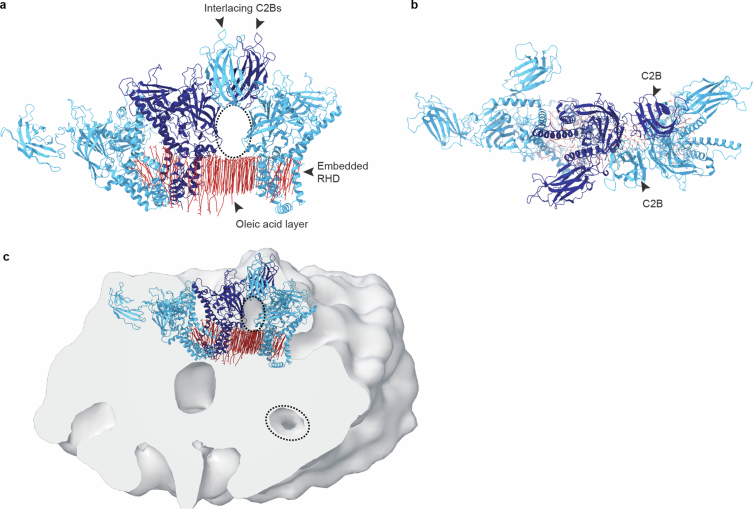
Structure prediction of trimeric *Pp*MCTP in the presence of fatty acids recapitulates the experimentally determined desmotubule coating assembly. AlphaFold3 enables structure prediction of proteins in the presence of selected ligands. When added in excess, the fatty acid ligand oleic acid approximates membrane-like chemical properties and improves prediction accuracy for membrane proteins. **a**, Side and **b**, top view of predicted trimeric *Pp*MCTP2 (monomers shown in alternating light and dark blue) with 140 oleic acid molecules (red) forming a layer around the reticulon homology domains (RHDs). C2B domains interlace as observed in the experimentally resolved desmotubule-coating assembly. Dotted ellipse marks unoccupied spot between membrane and interlaced C2Bs. C2A and the flexible C2A-B linker are excluded. **c**, Angled cross-section of the experimental map with docked trimeric AlphaFold3 model. Unoccupied spots underneath interlaced C2B domain pairs align well in the model and experimental map (dotted circles).

To elucidate the molecular composition of desmotubule-coating assemblies, we derived a list of candidate proteins from a proteome of PD-enriched cell wall fractions (Supplementary Table 1). Proteins predicted to localize to the ER and those with potential scaffolding functions were included, while those assigned to the secretory pathway were excluded. We predicted structures of the remaining 43 candidate proteins and generated synthetic density maps for comparison with experimental maps of the assembly and subsequently performed rigid body fitting. Among all tested candidates, exclusively Multiple C2 Domain and Transmembrane Proteins (MCTPs) matched the experimental data (Fig. 4, Extended Data Fig. 5 and Supplementary Video 5). MCTPs colocalize with PD markers in land plants and have been identified in the proteomes of PD fractions across species^6–13^. In *P. patens*, the MCTP family includes six homologues (*Pp*MCTPs; Extended Data Table 1 and Extended Data Fig. 6) with conserved domain topology, three of which are highly enriched in PD fractions (*Pp*MCTP1, 2 and 5). *Pp*MCTP1 and *Pp*MCTP5 have previously been localized to PD in vivo^11,12^. We now also tested the localization of *Pp*MCTP2 and *Pp*MCTP4 via transient expression in *Nicotiana benthamiana*. Both homologues appear ER localized with accumulation at cell–cell interfaces, where they colocalized with the plasmodesmal callose marker aniline blue (Extended Data Fig. 7). In flowering plants, the MCTP family is increasingly well characterized, with multiple homologues localizing to PD, where they are implicated in membrane tethering^10,22,42,43^. At the carboxy terminus, MCTPs contain a reticulon homology domain (RHD)^22,44^. RHDs are widely conserved across eukaryotes and function in ER shaping^45,46^, including in ER-containing cytoplasmic bridges in the insect germline^47^. Consistent with our structural data, genetic and biochemical studies have indicated that plant MCTPs can form higher-order assemblies in vivo^22,48^, with support from recent modelling studies implicating their RHDs in mediating intermolecular interactions^49^. Through structure prediction and assessment of multiple oligomeric configurations, we identified dimers as the most likely arrangement of *Pp*MCTPs, with interfaces formed by two central α-helices per monomer and their membrane-embedded RHDs (Extended Data Figs. 6 and 8). Given the high sequence conservation among *Pp*MCTPs (77% global identity, 94% at the dimer interface), heterodimerization appears likely. Predicted homo- and heterodimers are nearly identical in overall structure, differing primarily in the length of a glycine-rich linker connecting the amino-terminal C2A domain to C2B (the C2A–B linker domain), which ranges from 80 to 150 residues across family members (Extended Data Fig. 6 and Extended Data Table 1). In total, *Pp*MCTPs contain four C2 domains. C2B, C2C and C2D are positioned around the central dimer interface, while C2A is flexibly tethered via the extended C2A–B linker domain (Fig. 4a). To determine how MCTPs organize within the desmotubule-coating assembly, we excluded the positionally flexible C2A and its linker and fitted the N-terminally truncated dimers into the density map obtained by subtomogram averaging. These truncated dimer units fully account for the resolved helical assemblies coating desmotubules in situ (Fig. 4b and Extended Data Fig. 5), affirming MCTPs as their fundamental building blocks.

Although side chain interactions remain unresolved at this resolution, we identified potential contact sites of individual dimer units at the domain level. In the resolved assembly, C2B domains interlace with the C2B domains of adjacent dimers in the neighbouring helical wrap (Fig. 4c), while C2C and C2D domains contact adjacent dimers within the same wrap (Fig. 4d). The ability of C2 domains to mediate protein–protein interactions has been reported for other C2-containing proteins^50,51^. The deletion of C2C in *A. thaliana* MCTP4 disrupts PD association^22^, supporting its role in stabilizing MCTP assemblies at PD. MCTPs have recently been described to prevent ER membrane abscission at the developing cell plate during cytokinesis, thereby facilitating PD formation through incomplete cell division^22^. The densely packed assemblies resolved here may provide the structural basis for this function, stabilizing the desmotubule and shielding it against membrane severing (Fig. 4e). Supporting this, we observed that under ABA-induced callose deposition, MCTP assemblies remain intact and are internalized into the cell wall along with desmotubules, plasma membrane tethers and cytosolic sleeves (Figs. 2a,b and 3i). Severing occurs cytoplasm-proximal to the assembly, retaining it as well as the central desmotubule and plasma membrane in position within the sealed pore.

### Extended C2A domains as desmotubule–plasma membrane tethers

In mature, post-cytokinetic cells, MCTPs contribute to permeability control through a callose-independent mechanism^16^. Higher-order mutants of *At*MCTPs exhibit increased intercellular transport, and molecular dynamics simulations suggest that the C2B, C2C and C2D domains of *At*MCTP4 can interact with PI4P lipids in the plasma membrane. This lipid-mediated tethering has been proposed to modulate cytosolic sleeve diameter and thereby influence permeability independently of callose turnover^16^. While we cannot exclude membrane binding of these domains, we did not observe direct contacts between the plasma membrane and the surface of the resolved fraction of *Pp*MCTP assemblies, containing C2B, C2C and C2D domains (Fig. 4c,f). However, with theoretical lengths of 30–50 nm, fully extended C2A–B linkers are sufficiently long to bridge the cytosolic sleeve, allowing C2A binding at the plasma membrane. We therefore measured the distance between the assembly surface and the plasma membrane in GHL17 PD and found a maximum tether span of 26.4 nm (19.8 ± 4.2 nm, *n* = 72; Extended Data Fig. 1f). While C2A domains and their linkers are too small and flexible to be resolved by subtomogram averaging, we observed electron-dense material in individual tomograms extending from the assembly surface towards the plasma membrane, coinciding with plausible trajectories of extended C2A–B linker domains (Fig. 4f). These densities terminate at membrane-proximal positions consistent with C2A-mediated lipid binding, supporting a model in which C2A domains serve as the primary tethers linking desmotubule coats to the plasma membrane in *P. patens*.

### C2A–B linker domains are disordered polyampholytes

**Extended Data Fig. 9:**
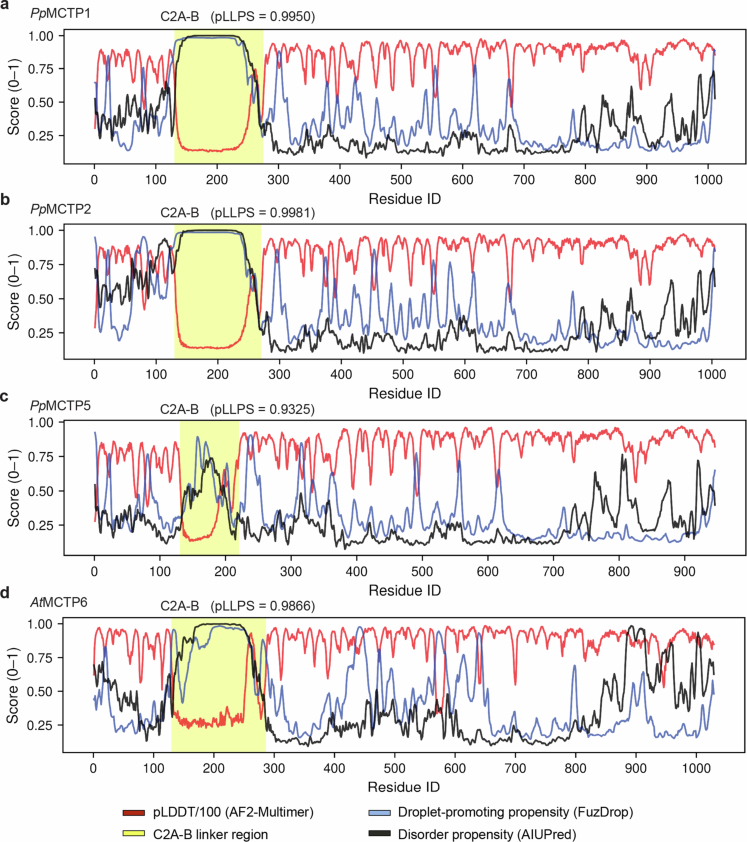
In silico profiling of sequence-encoded biophysical properties of full-length MCTPs. **a**, *Pp*MCTP1; **b**, *Pp*MCTP2; **c**, *Pp*MCTP5; **d**, Arabidopsis *At*MCTP6. Shared y-axis shows per-residue confidence scores from AlphaFold2-Multimer predictions (pLDDT/100, red), predicted droplet-promoting propensity (pDP) from FuzDrop (blue), and predicted disorder propensity scores from AIUPred (black). C2A-B linker domains are indicated in yellow. Liquid-liquid phase separation potentials (pLLPS, FuzDrop) of C2A-B linker domains are stated for each homolog.

Despite high overall conservation across the MCTP family, C2A–B linker domains are the most variable sequence regions, hinting at potential functional diversification. We initially noticed these linkers for their high glycine content at the sequence level. In subsequent structure predictions, they consistently scored below 50 in the predicted local distance difference test (pLDDT), an established predictor of intrinsic disorder^52^. Our in situ data indicate that C2A–B linkers populate cytosolic sleeves and are particularly concentrated at the narrow neck regions, prompting us to examine their potential role. Sequence analysis revealed that they are polyampholytes—that is, they contain a balanced distribution of oppositely charged residues interspersed with flexibility-promoting amino acids (Extended Data Fig. 6). Also referred to as sticker–spacer architectures, such motifs have been implicated in multivalent protein–protein interactions and phase-separated states^53^. Consistent with these properties, sequence-based prediction algorithms classify all *P. patens* C2A–B linkers as disordered and assign a high intrinsic propensity for liquid–liquid phase separation (LLPS propensity > 93%; Extended Data Fig. 9). While some MCTPs in flowering plants lack C2A domains, the majority of *Arabidopsis* homologues retain all four C2 domains (*At*MCTP2, 6, 7, 8, 9, 10, 14, 15 and 16) and harbour C2A–B linkers with polyampholyte characteristics and comparable disorder and LLPS predictions (Extended Data Fig. 9), indicating potential functional relevance across land plants.

## Discussion

In this work, we report native architectures of PD across tissues and physiological states in *P. patens* using cryo-ET. We recovered similar overall pore dimensions as described in classical transmission electron microscopy and freeze-substitution studies, while also uncovering previously unrecognized features. Specifically, we resolved how ABA signalling reduces intercellular permeability by inducing plasmodesmal closure coinciding with increased callose levels. While callose-mediated constriction of plasmodesmal apertures is a well-established regulatory mechanism, our data reveal an additional outcome: local remodelling of the cell wall downstream of ABA can function as an apoplasmic gate, capable of fully sealing channels and abolishing both cytosolic and membrane continuity between cells. In flowering plants, sustained ABA signalling underlies several physiological arrest states, including bud dormancy^54^, floral meristem arrest^38,55^ and seed dormancy^56^, all associated with reduced symplasmic connectivity. In *P. patens*, week-long ABA exposure similarly induces a symplasmically isolated ‘brood-cell’ state^57^. The pore architectures captured here after 17 h of ABA exposure may represent an early phase of brood-cell transition, characterized by plasmodesmal closure but preceding other developmental hallmarks of brood cells such as the thickening of bulk cell walls, or they may reflect a distinct PD-regulatory program. Consistent with this interpretation, we observed cell wall thickening specifically at PD neck regions following ABA treatment, without corresponding changes in the bulk cell wall. PD-localized cell wall bulging has also been reported following callose synthase induction in *Arabidopsis*, although whether it can result in complete channel sealing remained unresolved^58^. Short-term ABA-induced inhibition of cell-to-cell movement is reversible within 24 h of hormone removal in *P. patens*^40^, raising the question whether and how membrane continuity is re-established when callose plugs are degraded.

Across all imaged conditions, desmotubules were coated by modular helical protein assemblies with tethers connecting to the plasma membrane. Although desmotubule-associated particles and tethers have been described previously^19–21^, their molecular identity remained unresolved. By combining subtomogram averaging with proteome-scale structure prediction, we identified MCTPs as the likely building blocks of the desmotubule coat and their disordered C2A–B linker regions as likely candidates for the observed tethers. In mature gametophores, where MCTP assemblies and tethers span the full plasmodesmal length, reduced intercellular transport has been reported^30^, whereas the loss of MCTPs in *Arabidopsis* roots increases cell-to-cell conductivity^16^, illustrating that MCTP levels correlate with PD permeability. C2A–B linkers exhibit polyampholyte charge patterning, high predicted disorder and sequence-based propensity for multivalent interactions, raising the possibility that they contribute to permeability control within the cytosolic sleeve. Models invoking phase-separated protein networks as the molecular basis for selective permeability, analogous to FG-NUPs in the nuclear pore, have been discussed for PD^13,59,60^. The linker sequences described here are predicted to allow condensation but are more consistent with a mechanism based on non-specific electrostatic rather than hydrophobic interactions. Notably, plasmodesmal permeability has recently been shown to depend on solute charge in addition to size^61^. Polyampholyte linkers, by presenting a distribution of charged residues, may underlie such electrostatic selectivity. As MCTP assemblies are constitutively present, and sleeve diameter at neck regions is tuned by callose, the mechanisms could act synergistically. Low callose levels widen the sleeve and dilute the linker network, whereas high callose levels constrict the sleeve and increase local crowding (Extended Data Fig. 2).

Altogether, our data support a model in which MCTP dimers form helical coats around the desmotubule, anchored via RHDs, which contribute to membrane tubulation. Assemblies are stabilized by protein–protein interactions at the C2B, C2C and C2D domains. The C2A domains mediate tethering of the desmotubule to the plasma membrane, while polyampholyte C2A–B linkers populate the cytosolic sleeves, potentially forming dynamic diffusion barriers (Fig. 4g).

## Methods

### Preparation of *P. patens* tissues for cryo-ET

Culturing, sample preparation, cryo-fixation and focused ion beam (FIB) milling procedures are described in detail in ref. ^24^. Briefly, *P. patens* (Hedw.) Gransden ecotype was grown at 25 °C on BCDAT agar medium overlaid with cellophane, under 18 h white light / 6 h dark cycles. Small protonemata patches were detached using tweezers, submerged in freezing buffer (BCDAT liquid medium supplemented with Ficoll 400 dissolved in H_2_O to a final concentration of 20% (w/v)) and immediately high-pressure frozen (LEICA EM ICE, Leica Mikrosysteme GmbH). Gametophores were collected after four weeks by manually dissecting individual phyllids with fine scissors, followed by immediate immersion in freezing buffer and high-pressure freezing. The samples were vitrified using both the ‘waffle method’ and freezing in 3-mm carriers with a 100-µm-deep cavity, optimized for subsequent Serial Lift-Out FIB milling. GHL17 samples (*UBQ*::Pp*GHL17_1-mVenus* line, documented in ref. ^11^) were prepared as described for the WT.

### ABA treatment of *P. patens* protonemata

ABA stock solutions were prepared at 1,000× concentration (50 mM), dissolved in DMSO. Two-week-old WT cultures were treated by adding 7 ml of liquid BCDAT growth medium containing 50 µM ABA to the agar dishes. After 15 min of incubation, the solution was decanted. Seventeen hours after initial ABA exposure, the cells were high-pressure frozen as described previously.

### Cryo-ET data acquisition

Tilt series were collected using a Titan Krios G3i instrument at 300 kV, equipped with a Selectris X energy filter and a Falcon 4i camera (Thermo Fisher Scientific). Tilt series were recorded using the Tomography 5 software package (Thermo Fisher Scientific). A dose-symmetric tilt scheme was used with an angular increment of 3°. Magnification was set to ×64,000 (pixel size, 1.89 Å) with a dose rate of 3 e^−^ Å^−2^ per tilt, resulting in a total dose of 100 e^−^ Å^−2^ per tilt series. The target defocus ranged from −3.0 to −6.0 µm. A small dataset was collected on a Titan Krios G2 equipped with a Gatan post column energy filter and a K2 Summit (Gatan) camera. Here, tilt series were recorded at a magnification of ×42,000 (pixel size, 3.52 Å) using SerialEM v.3.9 (ref. ^62^).

### Tomogram reconstruction and segmentation

Tilt series were processed as described in ref. ^24^. Preprocessing steps including motion correction, removal of bad tilt images and dose filtering were performed in TOMOMAN (v.0.6.9)^63^. If a tilt series contained at least five platinum fiducials evenly distributed over the field of view, IMOD (v.4.11.25)^64^ fiducial tracking was employed for tilt-series alignment. Otherwise, AreTomo (v.1.3.3)^65^ was used for tilt-series alignment. Afterwards, 4× binned tomograms were reconstructed with the weighted back-projection algorithm implemented in IMOD. Membranes were segmented with MemBrain-Seg v.2 (ref. ^66^). While this software reliably traces intracellular membranes, it barely picked up PD membranes. Hence, the latest built-in model (v.10) was refined following the instructions available at https://teamtomo.org/membrain-seg/Usage/Training/. Furthermore, tilt series were imported into WARP (v.1.0.9)^67^. Membrane tethering densities within cytosolic sleeves were segmented using a convolutional neural network trained in EMAN v.2.99 (ref. ^68^). Because these features span only one to two pixels in width, training and segmentation were performed on bin2 tomograms that were denoised with cryo-CARE (v.0.1.1)^69^ and subsequently processed with IsoNet (v.0.3)^70^ employing the default parameters. For each tomogram, 1,000–1,200 negative reference regions were selected from areas containing cell wall, cytosol or non-PD membranes. Additionally, 30–50 positive reference areas were selected per tomogram, representing regions along the full PD channel. Positive references were manually annotated, serving as training input (training parameters: learnrate, 0.0001; iterations, 200; ncopy, 5; batch, 20; nkernel, 40,40,1; ksize, 9,7,5; poolsz, 2,1,1, box size, 128). While picking up tethers within cytosolic sleeves, the network failed to identify individual tethers at highly dense PD aperture regions. For those regions, an additional voxel density thresholding step was performed in Amira (v.2021.2, Thermo Fisher Scientific). All tomograms displayed in this work were denoised with cryo-CARE and visualized with IMOD (v.4.11.25). Segmentations were curated in Amira and jointly rendered in ChimeraX^71^.

### Cryo-electron tomogram analysis

PD dimensions were manually annotated with the contour tool in 3dmod (IMOD v.4.11.25)^64^ and the corresponding distances calculated in MATLAB (v.2022a, MathWorks). Data visualization and statistical analysis were performed in Python (v.3.11.5) using the libraries matplotlib, numpy, pandas, seaborn, scipy and statannotations. Statistical comparisons were conducted using two-sided Mann–Whitney–Wilcoxon tests, implemented via the statannotations.Annotator class. All comparisons were pre-planned against the WT control; no correction for multiple comparisons was applied. Supplementary Data 1 contains all statistical information including exact *P* values, means, medians, *U* statistics and standard deviations for each comparison. The script used for statistical analyses and data visualization is available via Zenodo (10.5281/zenodo.19224013)^72^.

### Subtomogram averaging of the desmotubule-coating assembly

For each desmotubule-coating assembly, a two-point contour was manually defined in 3dmod. The first point marked the beginning of the complex within the PD channel, and the second was placed at the channel opening towards the cytoplasm. In gametophore samples containing a single continuous assembly spanning the entire PD, a single long contour was defined from one channel opening to the other. Subtomograms (4× binned; voxel size, 7.6 Å; box size, 64 voxels) were extracted at 1-nm intervals along the line connecting these two points. Euler angles for *θ* and *ψ* were calculated such that the *z* axis of each subtomogram aligned parallel to the desmotubule contour axis; *φ* angles were randomized. Initial alignment was performed in STOPGAP (v.0.7.0)^73^ with shifts allowed only perpendicular to the axis of the PD channel without *φ* angle search. The reference for the first iteration was the average of the extracted subtomograms without alignment (Extended Data Fig. 4). Subsequently, the average of the current iteration was used for the next one. All references were filtered to a resolution of 35–40 Å.

For the GHL17 dataset, pseudo-symmetry expansion and a second alignment step in STOPGAP were performed. From every initial subtomogram position after the first alignment step, 11 new positions were defined by shifting 6 nm radially outward onto the desmotubule surface with an angular distance of ~33°. Euler angles were updated to align the subtomogram *z* axis perpendicular to the desmotubule surface using the STOPGAP script sg_motl_shift_and_rotate.m. Subtomograms were re-extracted at these oversampled positions (4× binned; voxel size, 7.6 Å; box size, 48 voxels) and aligned with exhaustive search over all three Euler angles, allowing a maximum shift of 11 nm. Following alignment, the subtomograms were distance-filtered by removing particles within 8 nm of one another, retaining only the subtomograms with the highest alignment score (the fast local correlation function in STOPGAP). Subsequently, the subtomograms were extracted in WARP (2× binned; voxel size, 3.8 Å; box size, 96 voxels) and subjected to 3D classification, refinement and postprocessing in RELION (v.3.0.5)^74^. The resolution of the GHL17-derived average was estimated according to the Fourier shell correlation (FSC_0.143_ ≈ 33 Å; Extended Data Fig. 4).

To enable comparison of desmotubule-coating assemblies across all datasets, subtomograms were extracted in WARP (voxel size, 7.6 Å; box size, 64 voxels) directly after the initial alignment in STOPGAP without symmetry expansion. These subtomograms were classified and aligned in RELION. During postprocessing, all averages were filtered to a resolution of 40 Å, enabling comparison (Fig. 3b–e).

### Proteome-derived list of desmotubule-coating candidates

The *P. patens* high-confidence PD proteome (HC300 (ref. ^11^)) was filtered for plasma-membrane- and ER-localized proteins containing at least one transmembrane or known membrane-binding domain. In addition, proteins annotated with potential scaffolding functions, as well as those lacking functional annotation in *P. patens*, were included. Transporters, hydrolytic enzymes, proteins with known functions in vesicle transport and proteins predicted to be secretory-pathway-targeted were excluded. This filtering yielded 43 candidate proteins for further assessment (Supplementary Table 1).

### Structure predictions of candidate proteins

Monomeric structures of all candidate proteins were predicted using AlphaFold v.2.3.1 (ref. ^75^). While monomers could in principle fit within the experimental density, their small size allowed a wide range of orientations, often resulting in biologically implausible placements (for example, membrane-associated domains facing the cytosolic sleeve) or complete burial within the map without accounting for key structural features. These ambiguities precluded reliable identification of monomeric fits. To address this, each protein was further evaluated individually using structural, biochemical and localization information from the literature, with the goal of identifying higher-order oligomers from a combination of monomeric PD proteins that could recapitulate the desmotubule-coating assembly. For proteins with documented oligomeric states, dimeric and trimeric structures were predicted using AlphaFold-Multimer v.2.3.1 (ref. ^76^). Predicted complexes with high confidence (0.8 × ipTM + 0.2 × pTM ≥ 0.60) were subjected to rigid-body fitting into the experimental density. For models containing extended disordered regions (pLDDT < 50), truncated variants retaining only structured domains and short interdomain linkers were generated. Non-MCTP candidates failed to align meaningfully with the density, producing fits that either occupied the volume non-specifically or conflicted with membrane topology. Adding repeating units of non-MCTP oligomers did not recover the observed organization of the coat (Extended Data Fig. 5a). In contrast, truncated *Pp*MCTP dimers aligned closely with the experimental density, recapitulating the periodic features of the desmotubule-coating assembly (Extended Data Fig. 5b,c). To quantify the fit, we generated a full model by tiling *Pp*MCTP dimers using rigid-body fitting, simulated a corresponding density map from the atomic model at the resolution of the experimental average and then calculated the cross-correlation between the synthetic and experimental maps (Extended Data Fig. 5d).

### Assessment of MCTP structure by structure predictions

To explore the structural basis of MCTP oligomerization, we predicted monomeric, dimeric and trimeric structures for each of the six *Pp*MCTPs using AlphaFold v.2.3.1 and AlphaFold-Multimer v.2.3.1. Dimeric assemblies were further modelled for all pairwise combinations of *Pp*MCTPs to evaluate the plausibility of homo- versus heterodimerization (Extended Data Fig. 6c). Only predictions with a weighted score of 0.8 × ipTM + 0.2 × pTM ≥ 0.6 were considered. The resulting structure predictions were further assessed on the basis of pLDDT and PAE scores. For trimer predictions, the flexible N terminus was truncated to reduce computing demands. AlphaFold3 prediction was performed on https://alphafoldserver.com/ (ref. ^77^).

### In silico profiling of sequence-encoded biophysical properties of full-length MCTPs

FuzDrop was used to predict propensity for droplet formation along full-length MCTP sequences and LLPS potential for the C2A–B linker domains^78^. We also applied AIUPred for disorder prediction^79^. pLDDT scores from AlphaFold2-Multimer (ranging from 1 to 100) were normalized by dividing by 100 to match the 0–1 scale of FuzDrop and AIUPred.

### Callose quantification

*Physcomitrium patens* samples, grown as described above, were incubated for 1 h in 0.1% (w/v) aniline blue dissolved in 0.1 M Sorenson’s Phosphate Buffer at pH 7.2. Imaging of three biological replicates per condition was performed using a Zeiss LSM 880 Airyscan with Objective C Plan-Apochromat ×40/1.2 Water DIC M27. *Z*-stacks were recorded at 1-µm distance steps, 1,024 × 1,024 frame size, 8 times averaging, 1.0 numerical zoom and pinhole 2.4 A.U., with excitation at 405 nm and an emission detection range of 420–520 nm. Quantification was performed in Fiji ImageJ2 (ref. ^80^). Analysis was applied according to ref. ^81^. Briefly, a rectangular region of interest, sufficiently sized to encompass the cell–cell interface, was set and saved for repeated use across samples. Measurements of ‘Area’ and ‘Integrated Density’ were recorded. Net signal values were calculated from the RawIntDens values at a cell–cell interface minus the RawIntDens value of the background signal. For the display in Extended Data Fig. 3, values were normalized by dividing by 1 × 10^6^.

### In vivo localization of *Pp*MCTPs

*Pp*MCTP2 and *Pp*MCTP4 were amplified via PCR and cloned into D-TOPO entry vectors, and subsequently a Gateway LR reaction was performed with the Gateway destination vector pVS331. The amplification primers were caccATGTCTGGTGGTCGTAAG (*Pp*MCTP2 forward), CTAGAGTATACGGTCCGATTGCG (*Pp*MCTP2 reverse), caccATGGCGCGCAAGCTCAT (*Pp*MCTP4 forward) and TCATAGAATACGGTCAGCCTGTGAAGG (*Pp*MCTP4 reverse).

The reaction with LR Clonase II (Invitrogen) was conducted according to the manufacturer’s instructions. *Agrobacterium* cultures (strain GV3101::pMP50 with the p19 helper plasmid for silencing suppression) were transformed with the expression plasmids. Fresh streaks of transformed agrobacteria were used to inoculate 4 ml of LB culture per plasmid, grown overnight at 28 °C with shaking. Cultures were pelleted (20800 rcf, 1 min), resuspended in 1.5 ml of infiltration buffer (50 µM acetosyringone, 0.01 M MgCl_2_, 0.01 M 2-(*N*-morpholino)ethanesulfonic acid) and incubated for 1 h in the dark. The concentration was adjusted to an OD_600_ of 0.1 by diluting with infiltration buffer. *N. benthamiana* leaves were infiltrated at the abaxial epidermis with a syringe after pricking with a needle five days prior to imaging. Expression was induced by spraying the abaxial sides of leaves with β-oestradiol (20 µM β-oestradiol, 0.1% (v/v) Tween-20) 24 h prior to imaging. 0.1% (w/v) aniline blue solution in 0.1 M Sorenson’s Phosphate Buffer at pH 7.2 was infiltrated 5 min before the leaf discs were mounted on a microscope slide. Single *Z*-slices were recorded using a Zeiss LSM 880 Airyscan with Objective C Plan-Apochromat ×40/1.2 Water DIC M27, focusing on the cell periphery. Cell-wide localization was recorded as *Z*-stacks with 1-µm *z*-distance steps. Aniline blue was excited at 405 nm with emission detected at 420–520 nm, and mVenus was excited at 514 nm with emission detected at 516–565 nm (scanning parameters: 524 × 524 frame size, 4 times averaging, 1.0 numerical zoom and pinhole 1.0 A.U.). Quantification was performed in Fiji ImageJ2 (ref. ^80^), and PD indices were calculated as described in ref. ^11^. The Fiji macro is available at https://github.com/SHaensch/2022_PD-index-quantification. Plasmids are available upon request.

### Reporting summary

Further information on research design is available in the Nature Portfolio Reporting Summary linked to this article.

## Supplementary information

Reporting Summary

Supplementary Video 1 Tomogram and 3D segmentation of a representative plasmodesma connecting two WT protonemata cells.

Supplementary Video 2 Tomogram and 3D segmentation of a representative plasmodesma connecting two WT gametophore phyllid cells.

Supplementary Video 3 Tomogram and 3D segmentation of a representative plasmodesma connecting two WT protonemata cells that were treated with 50 µM ABA for 17 h.

Supplementary Video 4 Tomogram and 3D segmentation of a representative plasmodesma connecting two protonemata cells constitutively overexpressing Pp*GHL17_1*.

Supplementary Video 5 Subtomogram average of the desmotubule-coating assembly and rigid body fitting of predicted *Pp*MCTP dimers.

Supplementary Table 1 Proteomics-derived list of desmotubule coating assembly candidate proteins with their corresponding *Arabidopsis* orthologues and functional annotations.

Supplementary Data 1 Statistical test results for all analyses, including sample sizes, descriptive statistics (mean, median and standard deviation), *U* statistics and *P* values.

## Source data

Source Data Fig. 2 Statistical source data.

Source Data Fig. 3 Statistical source data.

Source Data Extended Data Fig. 1 Statistical source data.

Source Data Extended Data Fig. 3 Statistical source data.

Source Data Extended Data Fig. 7 Statistical source data.

## Figures and Tables

**Extended Data Table 1:**
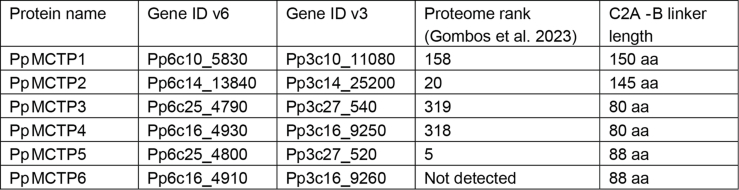
*Pp*MCTP gene identifiers and protein names

Gene identifiers, protein names, rank in proteome sorted by enrichment in PD fractions, and length of C2A-B linkers in amino acids. *Pp*MCTP1, 2, 3, 4, and 5 are found in the proteome with *Pp*MCTP1, 2 and 5 being most abundant.

## Data Availability

PD tomograms are available through the EM Data Bank for WT protonemata (EMD-56697 and EMD-57164), WT gametophores (EMD-57163 and EMD-57165), ABA-treated WT protonemata (EMD-57168 and EMD-57169) and GHL17 protonemata (EMD-57166 and EMD-57167). The desmotubule coat map is available under accession number EMD-55187. Source data are provided with this paper.

## Code availability

The script used for statistical analysis and data visualization is available via Zenodo at 10.5281/zenodo.19224013 (ref. ^72^).
