# Supplementary material for: In situ architecture of plasmodesmata in Physcomitrium patens resolved by cryo-electron tomography
Source: Nat Plants. 2026 May 14;12(5):1051–61. doi: 10.1038/s41477-026-02294-9 (PMC13197227; doi:10.1038/s41477-026-02294-9)
Supplement: Supplementary file 1 — Reporting Summary [file 41477_2026_2294_MOESM1_ESM.pdf]

nature portfolio

Corresponding author(s): Wolf B. Frommer, Wolfgang Baumeister, Marcel Dickmanns

Last updated by author(s): Mar 27, 2026

Reporting Summary

Nature Portfolio wishes to improve the reproducibility of the work that we publish. This form provides structure for consistency and transparency in reporting. For further information on Nature Portfolio policies, see our Editorial Policies and the Editorial Policy Checklist.

Statistics

For all statistical analyses, confirm that the following items are present in the figure legend, table legend, main text, or Methods section.

n/a	Confirmed	
☐	☒	The exact sample size (*n*) for each experimental group/condition, given as a discrete number and unit of measurement
☐	☒	A statement on whether measurements were taken from distinct samples or whether the same sample was measured repeatedly
☐	☒	The statistical test(s) used AND whether they are one- or two-sided<br>*Only common tests should be described solely by name; describe more complex techniques in the Methods section.*
☒	☐	A description of all covariates tested
☐	☒	A description of any assumptions or corrections, such as tests of normality and adjustment for multiple comparisons
☐	☒	A full description of the statistical parameters including central tendency (e.g. means) or other basic estimates (e.g. regression coefficient) AND variation (e.g. standard deviation) or associated estimates of uncertainty (e.g. confidence intervals)
☐	☒	For null hypothesis testing, the test statistic (e.g. *F*, *t*, *r*) with confidence intervals, effect sizes, degrees of freedom and *P* value noted<br>*Give P values as exact values whenever suitable.*
☒	☐	For Bayesian analysis, information on the choice of priors and Markov chain Monte Carlo settings
☒	☐	For hierarchical and complex designs, identification of the appropriate level for tests and full reporting of outcomes
☒	☐	Estimates of effect sizes (e.g. Cohen's *d*, Pearson's *r*), indicating how they were calculated

Our web collection on statistics for biologists contains articles on many of the points above.

Software and code

Policy information about availability of computer code

Data collection	Cryo-electron tomography tilt series were collected using Tomography 5 software (Thermo Fisher Scientific) and SerialEM v3.9. Fluorescence microscopy data was collected using Zen Black v14.0 and ZEN v2.3 SP1 FP3 (Zeiss).
Data analysis	Preprocessing of tomographic data was performed using TOMOMAN v0.6.9. Fiducial-based alignment was performed in IMOD v4.11.25, and fiducial-free alignment in AreTomo v1.3.3. Tomogram reconstruction was performed in IMOD v4.11.25. Denoising was performed using cryo-CARE v0.1.1 and IsoNet v0.3. Automated segmentation was performed with MemBrain-Seg v2 and EMAN v2.99. Curating and rendering were performed in Amira v2021.2 (Thermo Fisher Scientific) and ChimeraX v1.9. Tomograms were analysed using 3dmod in IMOD v4.11.25 and distance measurements were calculated in MATLAB v2022a (MathWorks). Data visualization and statistical analysis were performed in Python v3.11.5 using matplotlib v3.8.4, numpy v1.26.4, pandas v2.2.2, seaborn v0.11.2, scipy v1.13.1, and statannotations v0.5.0. Subtomogram averaging was performed using STOPGAP v0.7.0, WARP 1.0.9 and RELION v3.0.5. Protein structure predictions were generated using AlphaFold v2.3.1, AlphaFold-Multimer v2.3.1 and AlphaFold 3 Server (Google DeepMind, Isomorphic Labs). Rigid body fitting was performed in ChimeraX v1.9. In silico sequence analysis was performed using FuzDrop and AIUPred webservers. Fluorescence microscopy data was analyzed in Fiji ImageJ v2.17. Custom scripts were used for data handling and statistical analysis; these relied on standard Python scientific libraries as stated above and did not implement novel algorithms.

For manuscripts utilizing custom algorithms or software that are central to the research but not yet described in published literature, software must be made available to editors and reviewers. We strongly encourage code deposition in a community repository (e.g. GitHub). See the Nature Portfolio guidelines for submitting code & software for further information.

Data

Policy information about [availability of data](#)

All manuscripts must include a [data availability statement](#). This statement should provide the following information, where applicable:

- Accession codes, unique identifiers, or web links for publicly available datasets
- A description of any restrictions on data availability
- For clinical datasets or third party data, please ensure that the statement adheres to our [policy](#)

Data availability: Plasmodesmata tomograms are available through the EM Data Bank for WT protonemata (EMD-56697, EMD-57164), WT gametophores (EMD-57163, EMD-57165), ABA-treated WT protonemata (EMD-57168, EMD-57169), and GHL17 protonemata (EMD-57166, EMD-57167). The desmotubule coat map is available under accession EMD-55187.
Code availability: The script used for statistical analysis and data visualization is available on Zenodo (https://doi.org/10.5281/zenodo.19224013).

Research involving human participants, their data, or biological material

Policy information about studies with [human participants or human data](#). See also policy information about [sex, gender (identity/presentation), and sexual orientation](#) and [race, ethnicity and racism](#).

Reporting on sex and gender	n/a
Reporting on race, ethnicity, or other socially relevant groupings	n/a
Population characteristics	n/a
Recruitment	n/a
Ethics oversight	n/a

Note that full information on the approval of the study protocol must also be provided in the manuscript.

Field-specific reporting

Please select the one below that is the best fit for your research. If you are not sure, read the appropriate sections before making your selection.

☒ Life sciences ☐ Behavioural & social sciences ☐ Ecological, evolutionary & environmental sciences

For a reference copy of the document with all sections, see [nature.com/documents/nr-reporting-summary-flat.pdf](#)

Life sciences study design

All studies must disclose on these points even when the disclosure is negative.

Sample size	No statistical methods were used to predetermine sample sizes. Sample sizes were determined by the practical constraints of the cryo-ET workflow. Data were collected until 3 independent biological replicates per condition were obtained. Final sample sizes (n = 20 - 38 plasmodesmata per condition) are comparable to published cryo-ET studies of similar scope.
Data exclusions	Data from non-vitreous samples were excluded (pre-established criteria), as resulting artifacts preclude reliable analysis. From the remaining samples with proper preservation, no data were excluded. In some acquired tomographic volumes the structure of interest was not fully contained. Here, measurements requiring the complete structure (e.g. end-to-end length) were not obtained.
Replication	All biological replicates that yielded usable data produced consistent results.
Randomization	Experimental groups were predefined by genotype (WT, GHL17 OE) or treatment (ABA). Within each group, samples were drawn from independently grown cultures as described.
Blinding	Blinding during data collection was not feasible due to the nature of the imaging process. Data analysis was performed independently and in a blinded manner by two scientists.

Reporting for specific materials, systems and methods

We require information from authors about some types of materials, experimental systems and methods used in many studies. Here, indicate whether each material, system or method listed is relevant to your study. If you are not sure if a list item applies to your research, read the appropriate section before selecting a response.

Materials & experimental systems

n/a	Involved in the study
☒	☐ Antibodies
☒	☐ Eukaryotic cell lines
☒	☐ Palaeontology and archaeology
☒	☐ Animals and other organisms
☒	☐ Clinical data
☒	☐ Dual use research of concern
☐	☒ Plants

Methods

n/a	Involved in the study
☒	☐ ChIP-seq
☒	☐ Flow cytometry
☒	☐ MRI-based neuroimaging

Plants

Seed stocks	Physcomitrium patens (Hedw.) ecotype Gransden cultures were obtained from the International Moss Stock Center (IMSC, Freiburg, Germany). GHL17 cultures were acquired from the original authors who generated the moss line as described in the Methods section.
Novel plant genotypes	No novel plant genotypes were generated in this study.
Authentication	The GHL17 line was confirmed by PCR at the time of material transfer.
